# The Ethics of Human Embryo Editing via CRISPR-Cas9 Technology: A Systematic Review of Ethical Arguments, Reasons, and Concerns

**DOI:** 10.1007/s10730-024-09538-1

**Published:** 2024-09-20

**Authors:** Lindsay Wiley, Mattison Cheek, Emily LaFar, Xiaolu Ma, Justin Sekowski, Nikki Tanguturi, Ana Iltis

**Affiliations:** 1 Wake Forest University Center for Bioethics, Health and Society, Winston-Salem, USA; 2 Department of Communication Studies, University of Minnesota, Minneapolis, USA; 3 Department of Philosophy, Wake Forest University Center for Bioethics, Health and Society, Winston-Salem, USA

**Keywords:** Embryo editing, CRISPR, Genome editing

## Abstract

The possibility of editing the genomes of human embryos has generated significant discussion and interest as a matter of science and ethics. While it holds significant promise to prevent or treat disease, research on and potential clinical applications of human embryo editing also raise ethical, regulatory, and safety concerns. This systematic review included 223 publications to identify the ethical arguments, reasons, and concerns that have been offered for and against the editing of human embryos using CRISPR-Cas9 technology. We identified six major themes: risk/harm; potential benefit; oversight; informed consent; justice, equity, and other social considerations; and eugenics. We explore these themes and provide an overview and analysis of the critical points in the current literature.

## Introduction

Discussion of the prospect of human genome editing has lingered for decades, but for years it appeared to be only a distant possibility. Rapid developments, including the birth of twin girls in China whose embryos were reportedly edited, have created an urgent need to understand and address the ethical and regulatory issues human genome editing raises (Greely, 2019). Although many different tools have been developed and used for the purpose of gene editing, the most prominent one currently and hence the subject of this systematic review is Clustered Regularly Interspaced Short Palindromic Repeats through CRISPR-associated protein 9 (CRISPR-Cas9, often referred to as CRISPR). It provides numerous advantages over older technologies, in particular its increased efficiency, specificity, ease of use, and relative competitiveness with other similar technologies from a cost standpoint (Jinek et al., 2012). Some research using CRISPR has focused on editing genes rather than genomes, such as work that has been done on people with sickle cell disease (FDA, 2023). These interventions affect an individual’s somatic cells, but they do not change the person’s genome and thus would not be passed on to future offspring. Editing human embryos, on the other hand, involves changes in the genome that would be passed on in the future. Thus far, most human embryo editing using CRISPR has been strictly on embryos that would not be gestated and allowed to be born, and scientists generally have agreed that CRISPR is not yet safe enough to be used clinically to edit human genomes (Greely, 2019; Jinek et al., 2012). He Jiankui’s work in China was an exception. He’s report that he had edited the genomes of embryos to make them resistant to HIV and transferred the embryos for gestation, a process that resulted in the birth of twin girls as well as a third child, sounded alarm bells (Lovell-Badge, 2019).

This systematic review was to designed to analyze the biomedical and bioethics literature that addresses the use of CRISPR-Cas9 technology for human embryo editing to identify the ethical arguments and reasons that have been offered for and against heritable human genome editing as well as concerns raised. The literature we reviewed addressed four broad categories of potential applications of heritable human genome editing using CRISPR-Cas9 technology: to prevent fatal or serious genetic diseases such as Tay Sachs Disease and Huntington Disease; to enhance cognitive, physical, or moral capacities; to boost the immune system or confer immunity to particular conditions (as in the He Jiankui experiment); and to extend the lifespan of otherwise healthy individuals. We identified six major ethical themes and sixteen sub-themes. We discovered a range of views on this subject with many publications endorsing the use of this technology to edit human embryos, others rejecting its use, and most not taking a stance.

## Methods

This systematic review followed PRISMA reporting guidelines (Moher et al., 2015). The protocol is available upon request. The research question formulated for this review was: What are the ethical arguments, reasons, or concerns offered for and against the editing of human embryos using CRISPR-Cas9 technology in the bioethics and biomedical literature?

### Search

Our search was conducted on January 29, 2023 and included the use of three databases: PubMed, EmBase, and Web of Science. In PubMed, Web of Science, and EmBase, we used the search string: (human) AND ((embryo editing) OR (gene editing) AND (CRISPR) AND (ethics) to obtain titles and abstracts of relevant articles. This search was designed and conducted in consultation with a research librarian (Colleen Foy, MLIS). We included peer reviewed articles, book reviews, letters to the editor, opinions or editorials, and theses or dissertations consistent with published recommendations (Scherer & Saldanha, 2019) (Paez, 2017). We limited our search to English language publications that address human embryos. PubMed and EmBase searches consisted of date limits from 2014 to 2023 and Web of Science search consisted of date limits 2015–2023. A fully reproducible search strategy is presented in Appendix 1.

### Eligibility and Selection

**Fig. 1:**
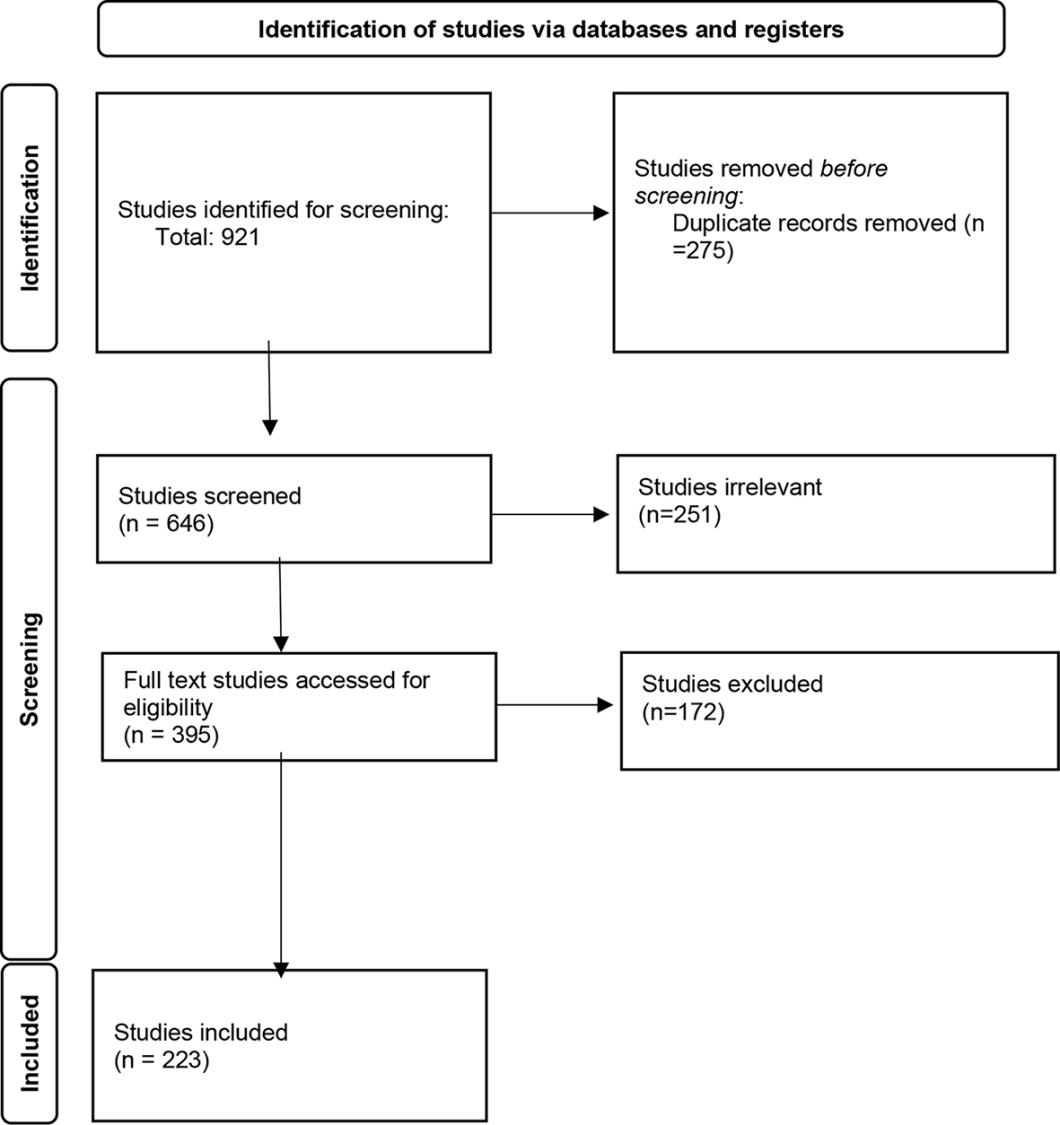
PRISMA Flow Chart (Page et al., 2021)

We developed inclusion and exclusion criteria (see Table 1) to guide screening of titles and abstracts as well as full texts of each publication. Six team members (LW, MC, EL, XM, JS, NT) participated in both screening phases and two team members screened each publication during each phase. Publications were excluded based on the highest-ranking exclusion criterion. All conflicts were resolved through consensus in team meetings and in consultation with ASI. After title and abstract screening, full texts were obtained and stored in Zotero. Full text screening results were recorded in Covidence. Figure 1 shows the screening process and results.

**Table 1:** Inclusion and exclusion criteria

Inclusion Criteria	Exclusion Hierarchy
1. Written in English 2. Available to us in full text 3. Has a primary focus on ethical arguments, reasons, or concerns associated with using CRISPR-Cas9 technology to edit human embryos or human genomes	1. Any publication not written or translated in English 2. Any publication not accessible to us online through the university library or via inter-library loan 3. Any publication that does not directly address human embryo editing or genome editing (e.g., addresses gene therapies) 4. Any publication that does not address any ethical arguments, issues, or concerns regarding the use of genome or embryo editing but rather only gives information 5. Any publication that does not have a primary focus on ethical arguments, reasons, or concerns associated with using CRISPR-Cas9 technology to edit human embryos or genomes

### Data Extraction

Data extraction responsibilities were divided equally among six team members (LW, MC, EL, XM, JS, NT). A data extraction tool was designed in Google Sheets. Two members of the team extracted data for each article and discrepancies were discussed between those two members along with a third member of the team and in consultation with ASI when necessary. Data extracted for each publication included: full citation; publication type; ethical argument(s), reason(s), or concern(s) regarding the use of CRISPR-Cas9 technology for human embryo editing mentioned; whether the author(s) argued for or against human embryo editing using CRISPR or remained neutral; the specific applications or uses of the technology mentioned; proposed solutions or approaches to addressing ethical issues; and the country listed for the first or corresponding author. We also included a separate section for notes to document other information we deemed important.

## Results

**Fig. 2:**
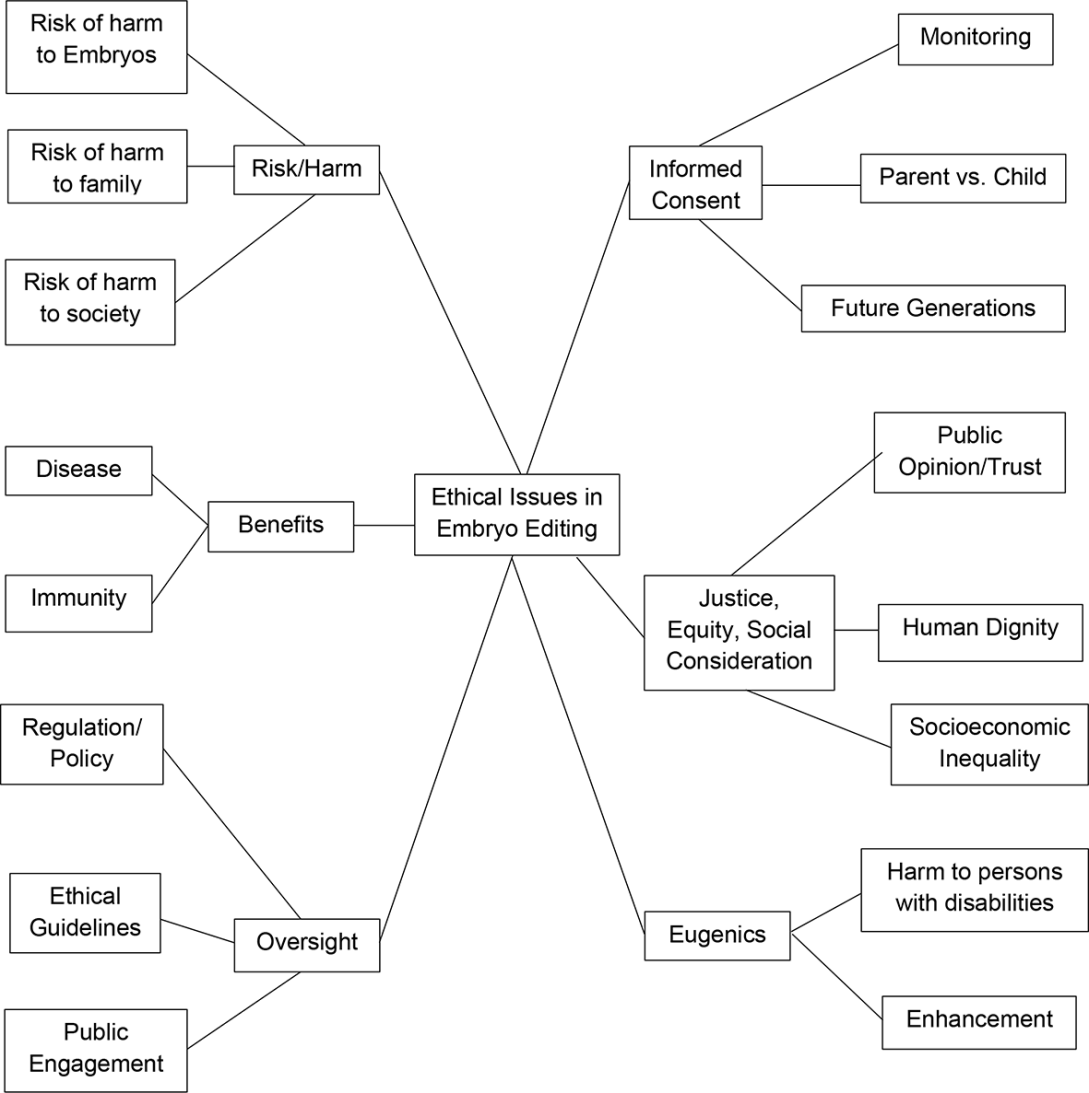
Themes

After full text screening, 223 publications were included in this systematic review (see Appendix 2 for table of included publications). We identified six primary categories of ethical reasons, concerns, or arguments: risk/harm (*n* = 178, 79.8%), potential benefit (*n* = 127, 56.9%), oversight (*n* = 196, 87.8%), informed consent (*n* = 86, 38.5%), justice, equity, and other social considerations (*n* = 80, 35.8%), and eugenics (*n* = 59, 26.4%). Analysis of each theme to identify more specific topics yielded sixteen subthemes, which are displayed in Fig. 2.

### Characteristics of Articles

Our literature search yielded 223 articles for inclusion, all published between 2014 and 2023. We found a high degree of geographic diversity based on the country of the first or corresponding author with 29 countries represented in our sample. More primary authors came from the United States (*n* = 104; 46.6%) than any other country, which was not surprising given that we limited our search to English language publications. Other well-represented countries included the United Kingdom (*n* = 20; 9.0%), Canada (*n* = 13; 5.8%), Germany (*n* = 9; 4.0%), Japan (*n* = 7; 3.1%), and China (*n* = 7; 3.1%). Our sample also included authors from the Netherlands, Sweden, Brazil, Costa Rica, Ireland, Israel, Jordan, Malaysia, New Zealand, Norway, and the Slovak Republic, which each only had one article. For 6 articles, we were unable to identify the country of the first or corresponding author.

The most prevalent type of article was a peer reviewed journal article (*n* = 131; 58.7%), followed by opinions/commentaries/editorials (*n* = 74; 33.2%), letters to the editor (*n* = 7; 3.1%), and book reviews (*n* = 3; 1.4%). The remaining 8 publications were marked Other and included 2 reviews, 2 magazine articles, 2 books, and one interview. Not surprisingly, we found many more publications that appeared after the He Jianku announcement in late 2018. Only one publication in our sample was from 2014. There were 12 (5.4%) from 2015, 14 (6.3%) from 2016, 31 from 2017 (13.9%), 21 from 2018 (9.4%), 66 (29.6%) from 2019, 42 (18.8%) from 2020, 24 (10.8%) from 2021, and 12 (5.4%) from 2022.

### Risks of Harm

Over 80% (*n* = 182; 81.6%) of included publications identified risk of harm or safety concerns associated with using CRISPR to edit human embryos. These fit into three broad categories: risks to the embryo, risks to family, and risks to society. Of particular concern was the safety and efficacy of the technology, specifically the possibility of off-target mutations and mosaicism (*n* = 140, 62.7%) and potential negative effects on future offspring/future generations (*n* = 91, 40.8%).

An overwhelming majority of the 182 articles addressed safety concerns associated with genetic errors or health implications. The high frequency of mutations jeopardizes the safety and efficacy of CRISPR germline editing, not only for the initially edited embryos but also for future offspring. Among the publications addressing health and safety concerns for future generations, the primary considerations were the emergence of new disease and adverse health effects. Other concerns for future generations, such as the need to monitor people for long-term effects, are addressed below under Informed Consent.

A total of 78 (34.9%) publications addressed the risk of harm to society. Concerns included cloning, generating or exacerbating health disparities, biological weapons and warfare, the effect on ethical views or norms in society, and division and potential threat to religious views. Some of these are addressed in more detail below.

### Potential Benefits

Over half of the publications reviewed (*n* = 126; 56.5%) addressed the potential benefits of CRISPR technology in embryo editing, including potential benefits to embryos, parents, and society as a whole. The potential benefits discussed fell into two major groups: prevent serious or fatal disease (*n* = 166; 74.4%) and boost immunity or create resistance to disease (*n* = 55; 24.7%).

### Oversight

The majority of publications reviewed (*n* = 196, 87.9%) mentioned oversight of human embryo editing. We identified three subcategories related to this theme: government regulation and policy development; development of professional or other ethical guidelines; and public engagement. In 94 (48.0%) publications, authors expressed concern that current laws, regulations, and oversight mechanisms are inadequate to govern embryo editing. Many publications described the need for additional work to promote ethical conduct related to the use of CRISPR technology. Many authors called for additional government regulation and policy development in the field of human embryo editing (*n* = 129, 65.8%) or ethical oversight and governance (*n* = 140, 71.4%). Additionally, many authors recommended development of professional (*n* = 86; 43.9%) or international (*n* = 58, 30.0%) ethical guidelines in response to perceived gaps, with a smaller percentage calling for a moratorium on human embryo editing (*n* = 17, 8.7%). Finally, over half of the publications reviewed called for public engagement (*n* = 108, 55.1%) on human embryo editing. Another 13 publications (6.6%) proposed solutions that did not fit into the aforementioned categories, such as intergenerational monitoring of those who may be genetically modified (Cwik, 2017; Shozi, 2020).

A total of 109 (48.9%) publications recommended increased public engagement to determine the future of human embryo editing. Public engagement means involving the general public as well as affected communities and other stakeholders in open and transparent discussion of potential research and applications of the technology as well as in developing and implementing oversight or regulation (Iltis et al., 2021). Some publications addressed the scope and purpose of public engagement regarding human embryo editing. For instance, Scheufele et al. (2021) describe public engagement as “processes and initiatives focused on enabling public participation in the responsible innovation and development of new technologies, including the management and assessment of technological risk” (p. 1). They identify seven goals for public engagement (avoid potential controversy; educate the public; build democratic capacity through deliberation; widen representation of voices; solicit input on value debates triggered by science; enable responsible innovation; shape policy” (pp. 2–4) and explore how these might inform a framework for public engagement on human embryo editing. Examples of the engagement they consider include consultations, collaborative exercises to identify policy pathways and solutions, and consensus conferences. However, we found that the term ‘public engagement’ often was used with little or no explanation regarding the nature or purpose of engagement. For instance, Hough and Ajetunmobi (2019) argued for a moratorium on germline editing “at least until such time as the international community, in dialogue with the public, has established a just and equitable genome-editing framework” (p. 343). Public engagement sometimes is proposed as a mechanism for increasing accountability and public trust in science, though its efficacy is debated (Petts, 2008; Wynne, 2006; Slack 2018; Crowder & Determeyer, 2019).

In 33 (14.8%) publications, authors held that public engagement regarding human embryo editing has been inadequate to date. Scheufele et al. (2021) offered a more in-depth analysis of why public engagement to date has been inadequate with respect to human embryo editing than most publications. These include the difficulty in developing and scaling engagement efforts to reach different types of stakeholders, the failure to incentivize scientists to pursue public engagement, and the disconnect between engagement efforts and policy-making.

### Informed Consent

Among the 93 (41.7%) publications that mentioned informed consent, we identified three areas of discussion: 73 (32.7%) addressed questions about the need to secure the informed consent of the parents or parents and the subsequent potential child; 60 (26.9%) raised concerns about the informed consent of descendants of genetically edited children; and 31 (13.9%) raised concerns about the need for informed consent in light of the likely desire to monitor the individuals with edited genomes throughout their lifetimes. Such long-term monitoring raises concerns about respect for the autonomy of future persons. Among the 93 publications that addressed informed consent, 18 (8.1%) mentioned all three informed consent issues.

### Justice, Equity, and Other Social Considerations

Over one-third (*n* = 80; 36.0%) of the publications reviewed mentioned justice, equity, and other social considerations as relevant to evaluating human embryo editing. Among these we found three sub-categories of concern: public opinion and trust (*n* = 35; 15.7%), socioeconomic inequity (*n* = 41; 18.4%), and human dignity (*n* = 58; 26.0%). Human dignity concerns focused primarily on the possibility that altering the human genome would change what it means to be human. Both the Universal Declaration on the Human Genome and Human Rights (United Nations General Assembly, 1948) and the Convention on Human Rights and Biomedicine (also known as the Oviedo Convention)(Council of Europe, 1997) cite this as a reason to reject human genome germline editing. Socioeconomic inequity concerns focused on the possibility that due to insurance limits and high costs, affluent individuals would have disproportionate access to human embryo editing. Finally, concerns regarding public opinion and trust appeared in 35 out of 223 articles (15.7%). These publications typically proposed increased public engagement to build trust in science and confidence that scientists would use and limit human embryo editing appropriately.

### Eugenics

Eugenics refers to beliefs and practices aimed at controlling reproduction in order to improve the characteristics of human populations (*Eugenics*, n.d.). Of the 223 publications reviewed, 60 (26.9%) addressed eugenics. Two major sub-themes emerged from these contributions: enhancement (*n* = 116, 52.0%) and disability (*n* = 28, 12.6%). Types of enhancement addressed include cognitive, physical, and moral/behavioral enhancement. Among the 28 articles addressing disability, some authors expressed concerns over the implications that embryo editing to correct or eradicate disabilities could have on people with disabilities (*n* = 20, 8.9%). Other authors advocated for the involvement of individuals with disabilities and disability rights groups in discussion of genome editing (*n* = 11, 4.9%). Some authors spoke positively of the possibility of editing embryos to eliminate the presence of disabilities (*n* = 4, 1.8%).

### Authors’ Interpreted Viewpoint

The research team analyzed each publication and interpreted the authors’ stances on human embryo editing using CRISPR technology. Articles were sorted into three categories based on this interpretation: endorse the use of CRISPR for human embryo editing in at least some circumstances, reject the use of CRISPR for human embryo editing, or neither endorse nor reject the use of CRISPR for human embryo editing. We found 76 (34.1%) endorsing the use of CRISPR for embryo editing under at least some circumstances, 30 (13.5%) rejecting it, and 117 (52.5%) neither endorsing nor rejecting the use of CRISPR technology for human embryo editing.

## Discussion

This systematic review reveals a wide range of ethical, policy, and social considerations regarding heritable human genome editing using CRISPR-Cas9. We examine key issues from our findings as they relate to three primary questions that many authors implicitly or explicitly considered and that often are the root of discussions regarding the future of human embryo editing: (1) What could go wrong? (2) What could go right? and (3) How can we prevent the bad and promote the good?

1. *What Could Go Wrong?*

Not surprisingly, concerns over the risks of physical harm associated with human germline editing, particularly the possibility of off-target mutations, dominated the literature. Editing errors can cause serious consequences such as DNA deletion, gene mutations, and deletion of entire chromosomes, and these changes will be passed on to future offspring (Zhang et al., 2015; Ledford, 2020). Additionally, embryo editing could result in mosaicism, meaning that the embryo would have some modified and some unmodified cells. Mosaicism can result in intellectual disabilities and delayed development, but the consequences in this context remain unknown and difficult to predict (Rubeis & Steger, 2018). One author noted that such harms affect not only the children born with editing-induced errors but their families, and especially women who often have disproportionate caregiving responsibilities (Labude et al., 2022).

In addition to concerns over physical risks, many publications pointed to social risks associated with misuse or abuse of CRISPR to create “designer babies’’ or to enhance physical, behavioral, or cognitive capacities. Often, authors explicitly or implicitly appealed to the controversial treatment/enhancement distinction to justify some applications of CRISPR and reject others. Disagreements regarding the treatment/enhancement distinction involve the plausibility of the distinction itself, whether it is morally or practically significant, where and how to draw the line between treatment and enhancement, and the implications of categorizing interventions on either side of the line (Anderson, 1989; Bess, 2010; Juengst & Moseley, 2019; Juengst, 1997; Parens, 1998). Despite these problems, in the literature we reviewed, applications to prevent disease, particularly “serious disease,” often were described as therapeutic or comparable to other medical treatments whereas potential applications to make someone “better than normal” or to provide a “capacity boost” were described as enhancements.

Discussion of the possibility of using CRISPR to enhance humans cognitively and physically is part of a broader debate regarding human enhancement, though the prospect of germline changes raises some unique questions. Views on human enhancement in general range from those who object to at least some enhancement methods or targets (e.g., Harris, 2011; Levy, 2007; Sandel, 2009; Shermer, 2008) to authors who argue that certain forms of enhancement are permissible (e.g., Agar, 2015; Douglas, 2011) or even morally obligatory (e.g., Crutchfield, 2020; Heinrichs & Stake, 2018; Savulescu & Persson, 2012) and perhaps essential to the survival of humanity (e.g., Persson & Savulescu, 2011). It is plausible that some advocates of general human enhancement would support enhancement via CRISPR although some might favor for other means, such as pharmaceutical or neurotechnological interventions. Among the publications we reviewed, there was some disagreement regarding the permissibility and implications of embryo editing to “enhance” humans. Some authors defended at least some enhancement applications, such as life span extension (Alexiou et al., 2020). Others, like Garland-Thomson, argue that it is impermissible to use CRISPR to enhance traits because of the potential consequences, such as losing our ability to enjoy the unexpected and interfering with evolution negatively (Garland-Thomson, 2020). Another objection to enhancement comes from Sparrow, who argues that turning to enhancement could lead to human obsolescence (Sparrow, 2019). His concern is that ever-evolving genetic “improvements” will result in humans who are constantly “outdated” or “older models” that routinely are considered not good enough, which he holds raises serious social and philosophical questions. Noting that many characteristics that would be the targets of enhancement, such as intelligence and athleticism, are multifactorial and, therefore, not appropriate targets of CRISPR technology, Benston holds that these are irrelevant to the human embryo editing debate and warns that focusing on these could undermine support for human embryo, slowing down progress on research that could provide important health benefits (Benston, 2017).

Some objections to using CRISPR for human enhancement were related to concerns regarding equity, justice, and fairness (e.g., de Melo-Martín, 2022; Thompson, 2019). Such concerns arise frequently when novel, expensive technologies emerge (e.g., Cavaliere, 2023; Morrissey & Walker, 2018; Perehudoff et al., 2016; Veatch, 1994). The hypothesis is that the wealthy would benefit disproportionately, widening the gap between the social classes and creating greater separation within and across societies. In the research ethics literature, discussions of these topics span from questions about who has and lacks access to research participation, the fair sharing of research burdens and benefits, the importance of inclusion and representation of diverse populations in research, exploitation, and post-trial access to decisions about which research areas do and do not receive funding and which interventions studied (Beauchamp et al., 2002; Fabi & Goldberg, 2022; Iltis, 2009; London, 2007; London & Kimmelman, 2019; MacKay & Saylor, 2020; Phillips, 2011; Sofaer & Strech, 2011; Walker et al., 2018; Wendler & Shah, 2015). Much of the literature we reviewed focused largely on the possibility of inequitable access to embryo editing technology and the implications for widening the gap between the rich and the poor (e.g., Bu, 2019; Lee, 2022; Lorenzo & Esquerda, 2019; Macintosh, 2019; Marchant, 2021; Rulli, 2019; Thaldar et al., 2020). Nevertheless, disparities in use might not always be fully explained by ability to pay. For instance, in at least some countries that cover or subsidize assisted reproductive technology costs, children who are conceived via reproductive technologies are on average born to mothers of higher socioeconomic status than children conceived naturally (Imrie et al., 2023). In light of these patterns, it is plausible that embryo editing technology might be disproportionately adopted by persons of higher socioeconomic status even if costs are covered such that cost might not explain disparate use.

Finally, research on and clinical applications of genetics and genomics are bound up with the history of eugenics, and some of the literature reviewed reflected worries that treating CRISPR as a tool for improving population health could result in it becoming a eugenic tool. While the term ‘eugenics’ often is associated with Nazi atrocities that included the widespread killing of human beings deemed to be inferior, the United States has its own history of eugenics programs that focused on controlling reproduction by persons deemed unfit so as to prevent the birth of “undesirable” children (Lombardo, 2008, 2011, 2019). An often-quoted sentiment expressed by Justice Oliver Wendell Holmes in *Buck v. Bell* (274 U.S. 200 (1927) the case in which the U.S. Supreme Court affirmed that Virginia’s law permitting the sterilization of people residing in institutions to promote health and the welfare of society was constitutional, captures the views that drove these programs: “Three generations of imbeciles are enough” *Buck v. Bell* (274 U.S. 200 (1927) at 207) The possibility of using CRISPR to edit embryos so that only children with the most socially desired characteristics are born or to prevent the birth of people with particular characteristics raises the specter of this history. It was not surprising to see the worry that embryo editing will result in eugenic practices expressed because existing practices designed to prevent the birth of children with various conditions or characteristics, namely prenatal screening and prenatal testing practices as well as preimplantation genetic diagnosis, have been linked to this history (Iltis, 2016; King, 1999; Mehlman, 2011; Selgelid, 2014; Thomas & Rothman, 2016).

Eugenic-based objections to preventing the birth of children with certain characteristics often arise in the disability literature (e.g., Eberl, 2022; Hubbard, 2010; Miceli & Steele, 2007; Raz, 2005). Two distinct approaches to disability emerged in the literature we reviewed. Some, whom we discuss in the next section, saw the possibility of reducing or eliminating disability as a positive aspect of this emerging technology. Others, however, objected to genome editing to eliminate disabilities. For some authors, disabilities themselves are not problems to be deleted from the human experience. People with disabilities experience their differences as disabilities because of the world around them (Espinoza & Tenorio, 2022; Oliver, 2018). For example, members of the Deaf community may see themselves as part of a culture that communicates differently from how hearing people communicate rather than as “disabled,” and they may believe that editing embryos to confer the ability to receive audible input is unwarranted (Katz, 2020; Padden & Humphries, 2020). (For a critique of the social construction of disability view, see Anastasiou & Kauffman, 2013; Terzi, 2004.) Some authors raised other disability-based critiques of eliminating disabilities through genetic technologies. Such practices: show disrespect toward people with disabilities, could lead to an increase in intolerance of persons with disabilities, could result in a decreased willingness to accommodate and support persons with disabilities (see, for example, Clifton, 2021; Eberl, 2022; Parens & Asch, 1999, 2003; Stahl, 2022; Stangl, 2010). Embryo editing to eliminate disabilities also could further marginalize people with or regarded as having disabilities and increase the stigma associated with having a disability or having a child with a disability (Doxzen & Halpern, 2020; Labude et al., 2022; Snure Beckman et al., 2019). Similar concerns have been expressed in the literature on prenatal diagnosis and preimplantation genetic diagnosis (Krahn, 2011; McCabe & McCabe, 2011; Watson, 2021).

The physical risks associated with genome editing underscore the importance of additional in vitro research on genome editing. Should it be used clinically, there is a pressing need for long-term follow-up to assess safety and efficacy. The social risks identified demonstrate the need not only for ongoing research but for robust public engagement and additional oversight and regulation as research progresses, as discussed below.

(2) What Could Go Right?

Many authors noted that, despite the risks, human genome editing offers significant potential benefits to future children, parents, and society. Among them, there remains disagreement over which potential benefits justify which risks, and how much risk we should tolerate to secure various potential benefits. Much of the literature emphasized the potential to edit human embryos to prevent serious and otherwise incurable diseases, such as cystic fibrosis, Duchenne’s Muscular Dystrophy, and Tay-Sachs Disease (e.g., Jedwab et al., 2020; Scheufele et al., 2017). For example, in one survey of clinical geneticists and certified genetic counselors in Japan, respondents generally supported editing genes in somatic cells to overcome serious genetic diseases, but just over 30% supported editing the germline to prevent a serious genetic disease, and over 95% of those surveyed opposed human embryo editing for non-serious diseases or would agree to it only if it was guaranteed to be successful, i.e., it had a 100% success rate (Taguchi et al., 2019).

In much of the literature we reviewed, where authors advocated for allowing embryo editing only for preventing or treating “serious” or “severe” diseases, a primary reason for this limitation appeared to be the view that only in those cases could the potential benefits justify the risks. This restriction also would prevent some of the potential applications of CRISPR that raise the more serious concerns about equity and enhancement that some publications raised. A critical issue in these discussions is what counts as a serious condition or what criteria a condition must meet to be sufficiently serious to justify embryo editing. We noted that although many publications referred to concepts such as “serious” or “severe” diseases, they typically did not define them or offer criteria for categorizing conditions or symptoms as serious enough to justify embryo editing. Different understandings of these distinctions would lead to different judgments about the permissibility of embryo editing for various conditions. This is an important omission in the literature given that there is significant disagreement even among genetics professionals regarding what counts as a serious condition (Chadwick et al., 1998, 2001; Wakefield & Conrad, 2020; Wertz & Knoppers, 2002).

An additional consideration in judging when the risks of embryo editing may be justified include the penetrance (the percentage of people with a particular genotype who exhibit a particular trait) and expressivity (the extent to which a person with a particular genotype expresses a particular trait) of a given genotype. If only a small percentage of people with a genotype express the phenotype targeted for action, then some might judge that the risks of genome editing are too high to justify it. Similarly, if a genotype results in a wide range of phenotypic expression with some much milder or less significant than others, different judgments about the justifiability of embryo editing risks may emerge.

Beyond incurable and often fatal conditions, there was less evidence of agreement on what conditions would be appropriate targets of embryo editing or the circumstances under which embryo editing is permissible, particularly over the long-term. For example, one study surveying 500 genetics professionals worldwide found more support for embryo editing for conditions that would be fatal in childhood (96.2%) or would shorten lifespan or put individuals at risk for sudden death (75.1%) than for conditions that did not have a “significant effect on life-span” (41.5%) (Armsby et al., 2019). Similarly, the degree of disability that would be associated with a condition was associated with the acceptability of embryo editing, with 79% supporting it when a condition would render a person severely disabled, versus moderately disabled (67.8%), and mildly disabled (37.7%) (Armsby et al., 2019). Some authors supported embryo editing to create immunity or resistance to disease (e.g., Cwik, 2020; Zimbelman, 2022). As reported by van Dijke et al. (2018), some hold that prevention through genome editing could be preferable to treatment. Any argument against embryo editing for “treatable” conditions requires further clarification of what it means for a condition to be treatable (see Bailey, 2009) and whether potentially debilitating and difficult treatments, such as chemotherapy, radiation therapy, and surgery, are preferable.

A few publications noted that in addition to benefiting future children who could be born healthy when they otherwise would have faced serious conditions, embryo editing might benefit parents (Snure Beckman et al., 2019; Williams, 2017). For instance, authors of a study that involved interviewing parents of children with aneuploidies, such as Down Syndrome, found that some “felt gene editing could reduce abortions of affected fetuses by giving families another option to lessen medical burden” (Snure Beckman et al., 2019, p. 327).

In addition to lowering burdens on families, some authors addressed the possibility that embryo editing aimed at preventing serious genetic conditions also could benefit societies through reduced healthcare and other costs and increased productivity (van Dijke et al., 2018). Some authors invoke the principle of procreative beneficence, which describes a duty to have and choose children who are expected to have the best life possible, to argue that human embryo editing to remove disease and disability ought to be pursued (Malek, 2010; Williams, 2017). In recent years, the question of whether prospective parents might have a moral obligation to select against disability in their offspring has piqued the attention of many prominent philosophers and bioethicists. A commitment to procreative beneficence would lead one to conclude that not only *may* embryo editing be used to eliminate disabilities, but that it *should* be used to eliminate disabilities and conditions that could result in a person living a life other than the best one possible. As discussed in the previous section, these views have been sharply criticized by many people with disabilities and disability scholars.

Proponents of new technologies who want to see research and implementation succeed often are interested in developing guidelines, regulations, and oversight to protect the integrity of the system and avoid scandals that could undermine the entire field and impede progress. We have seen this in other areas, such as vascularized composite allotransplantation, where pioneers in the field sought to have their work brought under the oversight umbrella of traditional solid organ transplantation systems (Cendales et al., 2011; Glazier, 2016). Thus, supporters of embryo editing because of its potential benefits might also support oversight, regulation, and guidance to facilitate progress, although they might advocate for different approaches than people who oppose or are more skeptical of embryo editing.

(3) How can we avoid the bad and promote the good?

Research on embryo editing is one type of human embryo research, which has been subject to different regulations, laws, and guidelines in different countries for several decades (Matthews & Morali, 2020). A critical reason that embryo research regulation has been complex and subject to different guidelines and laws is the existence of competing views of the moral significance of the human embryo (see, e.g., Delaney, 2023; George & Tollefsen, 2008; Harris, 1990; Khushf, 1997; Cahill, 1997; Warnock, 1984). As early as the 1980s, in the United States, the National Institutes of Health (NIH) determined that it would not accept proposals regarding germ-line interventions and would fund only gene editing research that involved somatic cell interventions (National Institutes of Health, 2019; Walters et al., 2021). In 2015, Dr. Francis Collins, then Director of NIH, cited the NIH decision not to accept germ-line intervention proposals as part of his rationale for opposing NIH funding for research on gene editing in human embryos (Walters et al., 2021). A renewable provision included in the Consolidated Appropriation Act of 2016 banned federal funding for any research that would intentionally create or modify a human embryo including heritable genetic modifications (Cohen & Adashi, 2016; Consolidated Appropriations Act, 2016). However, researchers in the US may conduct this work using private funding. Clinical applications of embryo editing will be subject to FDA regulatory oversight and approval in the US (Karagyaur et al., 2019). The legal status regarding embryo genome editing varies among countries. Some countries such as the United Kingdom, allow some research to proceed (Callaway, 2016), whereas Canada criminally banned embryo genome editing research (Knoppers et al. 2017).

Many publications reviewed noted that embryo editing raises ethical questions and has societal implications that existing regulations do not adequately address, creating a need for clear regulations, guidance, policy, or oversight of embryo editing to prevent harm and promote good. It is important to remember that regulations, oversight, and guidelines cannot prevent all wrongdoing. It is reported that Chinese guidelines in place at the time of He’s experiments prohibited research involving heritable genome editing aimed at reproductive purposes (see Li et al., 2019). Despite this, He pursued the work that led to the announcement of the birth of “CRISPR twins.” In January 2020, He was sentenced to three years in prison by the Chinese authorities for the “illegal medical practice” (Cyranoski, 2020). He’s work triggered a global outcry and condemnation from scientists, with some calling for a moratorium on human germline editing (Hough & Ajetunmobi, 2019). Shortly after He’s announcement, in March of 2019, an advisory committee formed by the World Health Organization (WHO) concluded that ‘‘it is irresponsible for anyone to proceed with human germline genome editing,’’ but stopped short of recommending a moratorium (Belluck, 2019, p. A1). In 2021, the Expert Advisory Committee on Developing Global Standards for Governance and Oversight of Human Genome Editing of the WHO released a report that included two documents: *Human Genome Editing: Recommendations* and *Human Genome Editing: A Framework for Governance* (Cohen et al., 2022). The committee reiterated its 2019 view and urged all those conducting or aware of research regarding human germline editing to engage with the committee immediately (World Health Organization, 2021). In addition, it recommended that the Director General of WHO “’make a policy statement to the effect that somatic and germline human genome editing research should only take place in jurisdictions with domestic policy and oversight mechanisms’” and discourage individuals from traveling to countries with less regulatory oversight to seek gene editing research or therapy (Cohen et al., 2022, p. 11, quoting WHO 2021). Although many argue for oversight and regulation, there remains a significant lack of clarity and consensus regarding what they should entail.

Research regulations and oversight policies often address critical ethical issues in the abstract and the challenge is applying them, particularly when research involves emerging technologies because there are more unknowns than usual. A special concern in human embryo editing is that embryos are not considered living human subjects according to many existing regulations and policies. Moreover, in the United States, even if they were, embryo research would not be subject to federal regulations governing human research at this time because such research cannot be federally funded by law and the federal human research regulations are binding only for federally funded research or research used to generate data to be used in seeking FDA approval. Nevertheless, even if one does not recognize human embryos as human beings to be protected by existing regulations or human subjects research ethics norms, were editing to be carried out in embryos intended for gestation, it would involve research on future persons. This raises serious questions about ethical obligations to future persons.

One particularly complex area in considering policies both for research and future clinical applications of human genome editing is how the widely accepted obligation to obtain informed consent would or should apply in the context of heritable human genome editing. Informed consent is typically required in both the clinical and research settings. Although standards vary regarding when consent is necessary and what is required for valid consent, typically it requires sharing relevant information, such as the potential benefits, risks, and alternatives, with authorized decision-makers and obtaining their free and voluntary permission to proceed. Ordinarily, parents authorize their children’s medical treatment and their participation in research. When minors are enrolled in research that will continue after they reach the age of majority, often age 18, they must be offered the opportunity to decide whether to remain in a study and give their own informed consent to continue participation. This could undermine the long-term monitoring of adults whose genomes were editing and their off-spring because such individuals could decline continued follow-up (Dickenson & Darnovsky, 2019).

Research participants routinely are told that they have the right to withdraw from research, and many guidelines and regulations stipulate this as a requirement for the ethical conduct of research. (e.g., Council for International Organizations of Medical Sciences, 2016; Protection of Human Subjects, 2018; World Medical Association, 2013). The irreversibility of genome editing raises questions about what it would mean to withdraw from a study. Parents who agreed to have their embryos edited could decide not to proceed with embryo transfer and not pursue pregnancy, effectively leaving a study that was meant to track outcomes in children. And, despite the view that it is important to monitor people born subsequent to genome editing over the long-term for safety and efficacy, parents could refuse to have a child followed in research (Dickenson & Darnovsky, 2019). Beyond refusing to be monitored, it is unclear what it would mean to have a right to withdraw from research involving genome editing given that there is likely no possibility of “undoing” the edits.

As gene editing research advances, a major question for oversight bodies will be when, if ever, it is safe enough to study in human embryos intended for gestation. Critical questions will include when, for what purposes, and in whom embryo editing may be studied. Proponents of the precautionary principle, which holds that a new technology may not be used until we can be reasonably certain that a technology is safe and does not introduce risk, will require a very high standard of proof. They prefer to avoid any risk that a technology introduces regardless of the costs of delaying, including the potential benefits of implementation (Cranor, 2004; Kopelman et al., 2004). Others will disagree, tolerating varying levels of unknown risk in light of the potential benefits. Determining who will make these judgments and how is an important part of developing policies, regulations, and oversight mechanisms for genome editing.

An additional important consideration that emerged in much of the literature we reviewed was the role of the public in assessing research on and applications of embryo editing. Calls for public engagement often arise in literature regarding emerging technologies and novel research areas (Adashi & Cohen, 2022; Bubela, 2006; Frith, 2023; Hurlbut et al., 2018; Iltis et al., 2021; Lander et al., 2019; Lovell-Badge et al., 2021; Matthews & Iltis, 2019; Sugarman et al., 2023). Such engagement can help to promote accountability and consideration of all stakeholders in developing guidelines and policies as well as help to build public trust in science. Despite frequent calls for public engagement, there has been little attention to defining it, identifying and justifying an account of the “public” or the “stakeholders” to be engaged, actively using effective practices to engage the public, articulating the specific purposes and goals of engagement, or clarifying how to use results to inform research and practice (Iltis et al., 2021). An additional problem is that authors sometimes appear to conflate public engagement with public communication or outreach. Engagement requires multi-way communication and information sharing, with all parties meaningfully contributing to a dialogue. Outreach and communication, on the other hand, involve primarily information delivery rather than active listening and consideration of other viewpoints (Iltis et al., 2021). While many authors in our review mentioned public engagement and the importance of public trust, many of the publications reviewed did not address these topics substantively. For instance, they offered no indication of who constitutes “the public,” what public engagement might entail, the goals of such engagement, or how it might inform and change research. Public engagement regarding human embryo editing thus far has been limited (e.g., Nuffield Council on Bioethics, 2018; National Academies, 2017; Ginsburg et al., 2021; Houtman et al., 2023; Thaldar et al., 2022; Conley et al., 2023; Iltis et al., 2021). Several author groups have recommended pathways toward improved public engagement regarding embryo editing (e.g., Iltis et al., 2021; Jasanoff et al., 2019; Sugarman et al., 2023). It remains unclear whether substantial public engagement efforts are forthcoming. While many scholars advocate for it, it is possible that some scientists might hesitate to engage the public out of fear of public reaction, such as a concern that it could lead to efforts to ban this research or to further restrict funding for it.

This systematic review has several limitations. First, although primary authors represented at least 15 countries and some publications included authors from multiple countries, we included only articles that were published in English. We may have missed themes that appear only in literature written in other languages. The language restriction likely reduced the geographic diversity of included publications. We also omitted 23 publications that were unavailable to us. In categorizing the authors’ stances toward human embryo editing, it is possible that we misinterpreted some positions despite using two reviewers per publication and discussing any conflicts with other team members. We limited our search to publications that addressed human embryo editing using CRISPR-Cas9 technology because this is the technology that has been widely discussed in recent years. However, other human embryo editing technologies are under investigation and we did not search for publications that addressed those (Gaj et al., 2016; Li et al., 2020). This review included publications found in only three databases and that appeared between 2014 and January 29, 2023. This is a rapidly developing research area and considerations introduced after our search date, if any, are missing. It is possible that we failed to identify some relevant publications despite working with a reference librarian to design and execute the search strategy. This review did not assess the quality or significance of the included publications because the goal was to generate a broad overview. Finally, we did not design our data extraction process to assess the intensity or significance of any ethical concerns that were identified in the literature.

## Conclusion

The embryo editing literature addresses a broad range of ethical, social, and regulatory issues. Many publications call for additional policy, regulation, oversight, ethics guidelines, public engagement, and pauses or moratoria on editing human embryos intended for gestation, reflecting a sense of urgency and concern regarding further implementation of human embryo editing. At the same time, much of the literature focuses on the potential promise human genome editing offers to improve health and prevent disease, with some authors raising concerns about hindering research and translation.

Preventing experiments such as the one performed by He Jiankui while facilitating high-quality research requires continued efforts to improve monitoring both from within and beyond the scientific community. It also requires greater clarity regarding the types of research and applications of human embryo editing that are and are not permissible and the conditions under which such work may be carried out. Developing policies, guidelines, and standards in this area requires robust public engagement and transparency regarding the goals of such engagement and plans for how information generated will be used.

## Appendix 1: Search Strategy

*Database*: **PubMed**.

*Search String*: (human) AND ((embryo editing) OR (genome editing)) AND (CRISPR) AND (ethics).

*Keywords*: human, embryo editing, genome editing, CRISPR, ethics.

*Limits*: English language publications, human species, 2014–2023.

*Database*: **EmBase**.

*Search String*: (human) AND ((embryo editing) OR (gene editing)) AND (CRISPR) AND (ethics).

*Limits*: English language publications, human, 2014–2023.

*Database*: **Web of Science**.

*Search String*: (human) AND ((embryo editing) OR (genome editing)) AND (CRISPR) AND (ethics).

*Keywords*: human, embryo editing, genome editing, CRISPR, ethics.

*Limits*: English language publications, 2015–2023.

### Appendix 2: Included Publications

**Table:**

Number	Citation
1	Adashi, E.Y., M.M. Burgess, S. Burall, I.G. Cohen, L.M. Fleck, J. Harris, S. Holm, et al. 2020. “Heritable Human Genome Editing: The Public Engagement Imperative.” *CRISPR J.* 3 (6): 434–39. 10.1089/crispr.2020.0049.
2	Adashi, E.Y., and I.G. Cohen. 2022. “CRISPR Immunity: A Case Study for Justified Somatic Genetic Modification?” *J Med Ethics* 48 (2): 83–85. 10.1136/medethics-2020-106838.
3	Adashi, E.Y., and I. Glenn Cohen. 2020. “Heritable Human Genome Editing the International Commission Report.” *JAMA* 324 (19): 1941–42. 10.1001/jama.2020.19059.
4	Al-Balas, Q.A.E., R. Dajani, and W.K. Al-Delaimy. 2020. “The Ethics of Gene Editing from an Islamic Perspective: A Focus on the Recent Gene Editing of the Chinese Twins.” *Sci Eng Ethics* 26 (3): 1851–60. 10.1007/s11948-020-00205-5.
5	Alexiou, A., P. Simou, F. Alexiou, S. Chatzichronis, and G.M. Ashraf. 2020. “Social and Ethical Impact of Advanced Artificial and Biological Enhancements.” *Open Public Health J.* 13 (1): 62–68. 10.2174/1874944502013010062.
6	Anegon, I., and T.H. Nguyen. 2016. “‘My Life Needs Editing’ (Mort Sahl) and Genome Editing Needs Ethics.” *Curr. Gene Ther.* 16 (1): 1–2. 10.2174/1566523216999160128110659.
7	Angrist, M., R. Barrangou, F. Baylis, C. Brokowski, G. Burgio, A. Caplan, C.R. Chapman, et al. 2020. “Reactions to the National Academies/Royal Society Report on Heritable Human Genome Editing.” *CRISPR J.* 3 (5): 332–49. 10.1089/crispr.2020.29106.man.
8	Annas, G.J. 2020. “Genome Editing 2020: Ethics and Human Rights in Germline Editing in Humans and Gene Drives in Mosquitoes.” *Am J Law Med* 46 (2–3): 143–65. 10.1177/0098858820933492.
9	Anton, R. 2016. “On Recent Advances in Human Engineering Provocative Trends in Embryology, Genetics, and Regenerative Medicine.” *Politics Life Sci* 35 (2): 54–68. 10.1017/pls.2016.17.
10	Araki, M., and T. Ishii. 2014. “International Regulatory Landscape and Integration of Corrective Genome Editing into in Vitro Fertilization.” *Reprod. Biol. Endocrinol.* 12 (1). 10.1186/1477-7827-12-108.
11	Araki, M., and T. Ishii. 2016. “Providing Appropriate Risk Information on Genome Editing for Patients.” *Trends Biotechnol.* 34 (2): 86–90. 10.1016/j.tibtech.2015.12.002.
12	Araujo, M. de. 2020. “The Ethics of Genetic Cognitive Enhancement: Gene Editing or Embryo Selection?” *Philosophies* 5 (3): 20. 10.3390/philosophies5030020.
13	Arguedas-Ramírez, G. 2020. “Ethics and Global Governance of Human Germline Genome Editing: The Problem of Techno-Scientific Colonialist Paternalism.” *CRISPR J.* 3 (2): 83–88. 10.1089/crispr.2019.0045.
14	Armsby, A.J., Y. Bombard, N.A. Garrison, B.L. Halpern-Felsher, and K.E. Ormond. 2019. “Attitudes of Members of Genetics Professional Societies Toward Human Gene Editing.” *CRISPR J.* 2 (5): 331–39. 10.1089/crispr.2019.0020.
15	Baik, Elizabeth S., Abraham Koshy, and Bruce W. Hardy. 2022. “Communicating CRISPR: Challenges and Opportunities in Engaging the Public.” *Prog Mol Biol Transl Sci* 188 (1): 171–93. 10.1016/bs.pmbts.2021.11.004.
16	Baxter, J. 2021. “When Is It Safe to Edit the Human Germline?” *Sci Eng Ethics* 27 (4): 43. 10.1007/s11948-021-00320-x.
17	Baylis, F. 2019. “Questioning the Proposed Translational Pathway for Germline Genome Editing.” *Nat Hum Behav* 3 (3): 200. 10.1038/s41562-019-0544-3.
18	Baylis, F., and L. Ikemoto. 2017. “The Council of Europe and the Prohibition on Human Germline Genome Editing.” *EMBO Rep.* 18 (12): 2084–85. 10.15252/embr.201745343.
19	Benston, 2017. “Everything in Moderation, Even Hype: Learning from Vaccine Controversies to Strike a Balance with CRISPR.” *J Med Ethics* 43 (12): 819–23. 10.1136/medethics-2016-103666.
20	Benston, S. 2022. “Walking a Fine Germline: Synthesizing Public Opinion and Legal Precedent to Develop Policy Recommendations for Heritable Gene-Editing.” *J Bioeth Inq* 19 (3): 421–31. 10.1007/s11673-022-10186-8.
21	Blasimme, A. 2019. “Why Include the Public in Genome Editing Governance Deliberation?” *AMA J Ethics* 21 (12): E1065–70. 10.1001/amajethics.2019.1065.
22	Bosley, K.S., M. Botchan, A. L. Bredenoord, D. Carroll, R. Alta Charo, E. Charpentier, R. Cohen, et al. 2015. “CRISPR Germline Engineering–the Community Speaks.” *Nat Biotechnol* 33 (5): 478–86. 10.1038/nbt.3227.
24	Braun, Matthias, and Peter Dabrock. 2018. “Mind the Gaps! Towards an Ethical Framework for Genome Editing.” *EMBO Rep* 19 (2): 197–200. 10.15252/embr.201745542.
25	Brokowski, C. 2018. “Do CRISPR Germline Ethics Statements Cut It?” *CRISPR J.* 1 (2): 115–25. 10.1089/crispr.2017.0024.
26	Brokowski, C., and M. Adli. 2019. “CRISPR Ethics: Moral Considerations for Applications of a Powerful Tool.” *J. Mol. Biol.* 431 (1): 88–101. 10.1016/j.jmb.2018.05.044.
26	Brokowski, C., and M. Adli. 2020. “Ethical Considerations in Therapeutic Clinical Trials Involving Novel Human Germline-Editing Technology.” *CRISPR J.* 3 (1): 18–26. 10.1089/crispr.2019.0051.
27	Brokowski, C., M. Pollack, and R. Pollack. 2015. “Cutting Eugenics out of CRISPR-Cas9.” *Ethics Biol. Eng. Med.* 6 (3–4): 263–79. 10.1615/EthicsBiologyEngMed.2016016260.
28	Brothers, K.B., M. Devereaux, and R.M. Sade. 2019. “Bespoke Babies: Genome Editing in Cystic Fibrosis Embryos.” *Ann. Thorac. Surg.* 108 (4): 995–99. 10.1016/j.athoracsur.2019.04.030.
29	Bu, Q. 2019. “Reassess the Law and Ethics of Heritable Genome Editing Interventions: Lessons for China and the World.” *Issues Law Med* 34 (2): 115–46.
30	Camporesi, S., and G. Cavaliere. 2016. “Emerging Ethical Perspectives in the Clustered Regularly Interspaced Short Palindromic Repeats Genome-Editing Debate.” *Personalized Med.* 13 (6): 575–86. 10.2217/pme-2016-0047.
31	Caplan, A. 2019. “Getting Serious about the Challenge of Regulating Germline Gene Therapy.” *PLoS Biol* 17 (4): e3000223. 10.1371/journal.pbio.3000223.
32	Capps, B., R. Chadwick, Y. Joly, J.J. Mulvihill, T. Lysaght, and H. Zwart. 2017. “Falling Giants and the Rise of Gene Editing: Ethics, Private Interests and the Public Good Ruth Chadwick.” *Hum. Genomics* 11 (1). 10.1186/s40246-017-0116-4.
33	Carroll, D., and R.A. Charo. 2015. “The Societal Opportunities and Challenges of Genome Editing.” *Genome Biol.* 16 (1). 10.1186/s13059-015-0812-0.
34	Chneiweiss, H. 2018. “The Tsunami Named CRISPR/Cas9.” *Rev Neurol (Paris)* 174 (7–8): 487–88. 10.1016/j.neurol.2018.04.006.
35	Chneiweiss, H., F. Hirsch, L. Montoliu, A.M. Müller, S. Fenet, M. Abecassis, J. Merchant, et al. 2017. “Fostering Responsible Research with Genome Editing Technologies: A European Perspective.” *Transgenic Res.* 26 (5): 709–13. 10.1007/s11248-017-0028-z.
36	Cohen, I. Glenn, Jacob S. Sherkow, and Eli Y. Adashi. 2022. “Handle with Care: The WHO Report on Human Genome Editing.” *Hastings Cent Rep* 52 (2): 10–14. 10.1002/hast.1350.
37	Cohen, J. 2019. “Crossing the Line.” *Sci.* 366 (6465): 562–65. 10.1126/science.366.6465.562.
38	Coller, B.S. 2019. “Ethics of Human Genome Editing.” *Annu. Rev. Med.* 70 ((Coller B.S., collerb@rockefeller.edu) Allen and Frances Adler Laboratory of Blood and Vascular Biology, Rockefeller University, New York, NY, United States): 289–305. 10.1146/annurev-med-112717-094629.
39	Cribbs, A.P., and S.M.W. Perera. 2017. “Science and Bioethics of CRISPR-CAS9 Gene Editing: An Analysis towards Separating Facts and Fiction.” *Yale J. Biol. Med.* 90 (4): 625–34.
40	Cwik, B. 2017. “Designing Ethical Trials of Germline Gene Editing.” *N Engl J Med* 377 (20): 1911–13. 10.1056/NEJMp1711000.
41	Cwik, B. 2020. “Responsible Translational Pathways for Germline Gene Editing?” *Curr. Stem. Cell Rep.* 6 (4): 126–33. 10.1007/s40778-020-00179-x.
42	Darnovsky, M., and K. Hasson. 2020. “CRISPR’s Twisted Tales: Clarifying Misconceptions about Heritable Genome Editing.” *Perspect Biol Med* 63 (1): 155–76. 10.1353/pbm.2020.0012.
43	Davies, K., and B.M. Knoppers. 2019. “From Poetry to Policy: An Interview with Bartha Maria Knoppers.” *CRISPR J.* 2 (5): 249–52. 10.1089/crispr.2019.29071.bkn.
44	Davies, K. 2019. “Walk the Line: Debating a Germline Editing Moratorium.” *CRISPR J* 2: 74–76. 10.1089/crispr.2019.29052.kda.
45	Davies, K., and K.E. Davies. 2020. “Highway to HHGE: An Interview with Dame Kay E. Davies.” *CRISPR J* 3 (5): 325–31. 10.1089/crispr.2020.29104.kda.
46	Dayan, F. 2019. “CRISPR Cas-9 Genome Editing and Islam: A Religious Perspective.” *Bangladesh J. Med. Sci.* 18 (1): 7–13. 10.3329/bjms.v18i1.39540.
47	Dayan, F. 2020. “Ethico-Legal Aspects of Crispr Cas-9 Genome Editing: A Balanced Approach.” *Bangladesh J. Med. Sci.* 19 (1): 11–16. 10.3329/bjms.v19i1.43869.
48	De Melo-MartíN, I., 2022. “Reproductive Embryo Editing: Attending to Justice.” *Hastings Cent Rep* 52 (4): 26–33. 10.1002/hast.1406.
49	De Miguel Beriain, I. 2017. “Legal Issues Regarding Gene Editing at the Beginning of Life: An EU Perspective.” *Regen. Med.* 12 (6): 669–79. 10.2217/rme-2017-0033.
50	De Wert, G., B. Heindryckx, G. Pennings, A. Clarke, U. Eichenlaub-Ritter, C.G. Van El, F. Forzano, et al. 2018. “Responsible Innovation in Human Germline Gene Editing: Background Document to the Recommendations of ESHG and ESHRE.” *Eur. J. Hum. Genet.* 26 (4): 450–70. 10.1038/s41431-017-0077-z.
51	Dickenson, D., and M. Darnovsky. 2019. “Did a Permissive Scientific Culture Encourage the ‘CRISPR Babies’ Experiment?” *Nat. Biotechnol.* 37 (4): 355–57. 10.1038/s41587-019-0077-3.
52	DiEuliis, D., and J. Giordano. 2018. “Gene Editing Using CRISPR/Cas9: Implications for Dual-Use and Biosecurity.” *Protein Cell* 9 (3): 239–40. 10.1007/s13238-017-0493-4.
53	Dijke, I. van, L. Bosch, A.L. Bredenoord, M. Cornel, S. Repping, and S. Hendriks. 2018. “The Ethics of Clinical Applications of Germline Genome Modification: A Systematic Review of Reasons.” *Hum Reprod* 33 (9): 1777–96. 10.1093/humrep/dey257.
54	Doudna, J. 2015. “Perspective: Embryo Editing Needs Scrutiny.” *Nature* 528 (7580): S6. 10.1038/528S6a.
55	Doudna, J.A. 2020. “The Promise and Challenge of Therapeutic Genome Editing.” *Nature* 578 (7794): 229–36. 10.1038/s41586-020-1978-5.
56	Douglas, T., and K. Devolder. 2022. “Gene Editing, Identity and Benefit.” *Philos. Q.* 72 (2): 305–25. 10.1093/pq/pqab029.
57	Doxzen, K., and J. Halpern. 2020. “Focusing on Human Rights: A Framework for CRISPR Germline Genome Editing Ethics and Regulation.” *Perspect Biol Med* 63 (1): 44–53. 10.1353/pbm.2020.0003.
58	Eissenberg, J.C. 2021. “In Our Image: The Ethics of CRISPR Genome Editing.” *Biomol Concepts* 12 (1): 1–7. 10.1515/bmc-2021-0001.
59	Evans, J.H. 2021. “Setting Ethical Limits on Human Gene Editing after the Fall of the Somatic/Germline Barrier.” *Proc. Natl. Acad. Sci. U. S. A.* 118 (22). 10.1073/pnas.2004837117.
60	Evans, N.G., and J.D. Moreno. 2015. “Children of Capital: Eugenics in the World of Private Biotechnology.” *Ethics Biol. Eng. Med.* 6 (3–4): 285–97. 10.1615/EthicsBiologyEngMed.2016016594.
61	Evitt, N.H., S. Mascharak, and R.B. Altman. 2015. “Human Germline CRISPR-Cas Modification: Toward a Regulatory Framework.” *Am J Bioeth* 15 (12): 25–29. 10.1080/15265161.2015.1104160.
62	Feeney, O. 2019. “Editing the Gene Editing Debate: Reassessing the Normative Discussions on Emerging Genetic Technologies.” *NanoEthics* 13 (3): 233–43. 10.1007/s11569-019-00352-5.
63	Filip, L. 2021. “Genetic Enhancement, TED Talks and the Sense of Wonder.” *Med Humanit*, 47(2): 210–218. 10.1136/medhum-2020-012051.
64	Flescher, A. 2022. “The Virtue of Mortality.” *J. Relig. Ethics* 50: 361–385. 10.1111/jore.12403.
65	Fogleman, S., C. Santana, C. Bishop, A. Miller, and D.G. Capco. 2016. “CRISPR/CAS9 and Mitochondrial Gene Replacement Therapy: Promising Techniques and Ethical Considerations.” *Am. J. Stem Cells* 5 (2): 39–52. https://www.ncbi.nlm.nih.gov/pmc/articles/PMC5043096/
66	Friedmann, T. 2016. “An ASGCT Perspective on the National Academies Genome Editing Summit.” *Mol. Ther.* 24 (1): 1–2. 10.1038/mt.2015.228.
67	Gabel, I., and J. Moreno. 2019. “Genome Editing, Ethics, and Politics.” *AMA J Ethics* 21 (12): E1105–10. 10.1001/amajethics.2019.1105.
68	Garland-Thomson, R. 2020. “How We Got to CRISPR: The Dilemma of Being Human.” *Perspect Biol Med* 63 (1): 28–43. 10.1353/pbm.2020.0002.
69	Germani, F., S. Wäscher, and N. Biller-Andorno. 2021. “A CRISPR Response to Pandemics?: Exploring the Ethics of Genetically Engineering the Human Immune System.” *EMBO Rep.* 22 (3). 10.15252/embr.202052319.
70	“Germline Gene-Editing Research Needs Rules.” 2019. *Nature* 567 (7747): 145. 10.1038/d41586-019-00788-5.
71	Getz, L.J., and G. Dellaire. 2020. “Back to Basics: Application of the Principles of Bioethics to Heritable Genome Interventions.” *Sci Eng Ethics* 26 (5): 2735–48. 10.1007/s11948-020-00226-0.
72	Glannon, W. 2020. “Altered Inheritance: CRISPR and the Ethics of Human Genome Editing.” *Bioethics* 34 (5): 555–56. 10.1111/bioe.12729.
73	Gómez-Tatay, L., J.M. Hernández-Andreu, and J. Aznar. 2017. “Mitochondrial Modification Techniques and Ethical Issues.” *J. Clin. Med.* 6 (3). 10.3390/jcm6030025.
74	Gostimskaya, I. 2022. “CRISPR-Cas9: A History of Its Discovery and Ethical Considerations of Its Use in Genome Editing.” *Biochemistry (Mosc)* 87 (8): 777–88. 10.1134/S0006297922080090.
75	Grant, E.V. 2016. “FDA Regulation of Clinical Applications of CRISPR-CAS Gene-Editing Technology.” *Food Drug Law J* 71 (4): 608–33.
76	Greely, H.T. 2019. “CRISPR’d Babies: Human Germline Genome Editing in the ‘He Jiankui Affair.’” *J. Law Biosci.* 6 (1): 111–83. 10.1093/jlb/lsz010.
77	Greely, H.T. 2019. “Human Germline Genome Editing: An Assessment.” *CRISPR J.* 2 (5): 253–65. 10.1089/crispr.2019.0038.
78	Greenfield, A. 2018. “Carry on Editing.” *Br. Med. Bull.* 127 (1): 23–31. 10.1093/bmb/ldy020.
79	Greenfield, A. 2021. “Making Sense of Heritable Human Genome Editing: Scientific and Ethical Considerations.” *Prog. Mol. Biol. Transl. Sci.* 182: 1–28. 10.1016/bs.pmbts.2020.12.008.
80	Gross, M. 2015. “Bacterial Scissors to Edit Human Embryos?” *Curr. Biol.* 25 (11): R439–42. 10.1016/j.cub.2015.05.027.
81	Gumer, J.M. 2019. “The Wisdom of Germline Editing: An Ethical Analysis of the Use of CRISPR-Cas9 to Edit Human Embryos.” *New Bioeth* 25 (2): 137–52. 10.1080/20502877.2019.1606151.
82	Guttinger, S. 2018. “Trust in Science: CRISPR-Cas9 and the Ban on Human Germline Editing.” *Sci Eng Ethics* 24 (4): 1077–96. 10.1007/s11948-017-9931-1.
83	Gyngell, C. 2017. “Gene Editing and the Health of Future Generations.” *J. R. Soc. Med.* 110 (7): 276–79. 10.1177/0141076817705616.
84	Halpern, J., S. E. O’Hara, K.W. Doxzen, L.B. Witkowsky, and A.L. Owen. 2019. “Societal and Ethical Impacts of Germline Genome Editing: How Can We Secure Human Rights?” *CRISPR J* 2 (5): 293–98. 10.1089/crispr.2019.0042.
85	Hampton, T. 2016. “Ethical and Societal Questions Loom Large as Gene Editing Moves Closer to the Clinic.” *JAMA* 315 (6): 546–48. 10.1001/jama.2015.19150.
86	Harper, J.C., and G. Schatten. 2019. “Are We Ready for Genome Editing in Human Embryos for Clinical Purposes?” *Eur. J. Med. Genet.* 62 (8): 103682. 10.1016/j.ejmg.2019.103682.
87	Harris, J. 2015. “Edited Genes and Slippery Slopes: Dilemmas in the Ethics of Gene Editing.” *Ethics Biol. Eng. Med.* 6 (3–4): 281–84. 10.1615/EthicsBiologyEngMed.2016016475.
88	Heidari, R., D.M. Shaw, and B.S. Elger. 2017. “CRISPR and the Rebirth of Synthetic Biology.” *Sci. Eng. Ethics* 23 (2): 351–63. 10.1007/s11948-016-9768-z.
89	Hershlag, A. and S.L. Bristow. 2018. “Editing the Human Genome: Where ART and Science Intersect.” *J Assist Reprod Genet* 35 (8): 1367–70. 10.1007/s10815-018-1219-0.
90	Hirsch, F., C. Lemaitre, H. Chneiweiss, and L. Montoliu. 2019. “Genome Editing: Promoting Responsible Research.” *Pharm. Med.* 33 (3): 187–91. 10.1007/s40290-019-00276-1.
91	Hirsch, F., R. Iphofen, and Z. Koporc. 2019. “Ethics Assessment in Research Proposals Adopting CRISPR Technology.” *Biochem Med (Zagreb)* 29 (2): 020202. 10.11613/BM.2019.020202.
92	Hofmann, B. 2018. “The Gene-Editing of Super-Ego.” *Med Health Care Philos* 21 (3): 295–302. 10.1007/s11019-018-9836-z.
93	Hollister, B.M., M.C. Gatter, K.E. Abdallah, A.J. Armsby, A.J. Buscetta, Y.J.J. Byeon, K.E. Cooper, et al. 2019. “Perspectives of Sickle Cell Disease Stakeholders on Heritable Genome Editing.” *CRISPR J.* 2 (6): 441–49. 10.1089/crispr.2019.0034.
94	Hough, S. H., and A. Ajetunmobi. 2019. “A CRISPR Moratorium Isn’t Enough: We Need a Boycott.” *CRISPR J* 2 (6): 343–45. 10.1089/crispr.2019.0041.
95	“How to Respond to CRISPR Babies.” 2018. *Nature* 564 (7734): 5. 10.1038/d41586-018-07634-0.
96	Howard, H.C., C.G. Van El, F. Forzano, D. Radojkovic, E. Rial-Sebbag, G. De Wert, P. Borry, and M.C. Cornel. 2018. “One Small Edit for Humans, One Giant Edit for Humankind? Points and Questions to Consider for a Responsible Way Forward for Gene Editing in Humans.” *Eur. J. Hum. Genet.* 26 (1): 1–11. 10.1038/s41431-017-0024-z.
97	Hynes, R.O., B.S. Coller, and M. Porteus. 2017. “Toward Responsible Human Genome Editing.” *JAMA* 317 (18): 1829–30. 10.1001/jama.2017.4548.
98	Hyun, I., and C. Osborn. 2017. “Query the Merits of Embryo Editing for Reproductive Research Now.” *Nat. Biotechnol.* 35 (11): 1023–25. 10.1038/nbt.4000.
99	Isa, N.M., N.A. Zulkifli, and S. Man. 2020. “Islamic Perspectives on CRISPR/Cas9-Mediated Human Germline Gene Editing: A Preliminary Discussion.” *Sci Eng Ethics* 26 (1): 309–23. 10.1007/s11948-019-00098-z.
100	Ishii, T. 2015. “Germline Genome-Editing Research and Its Socioethical Implications.” *Trends Mol. Med.* 21 (8): 473–81. 10.1016/j.molmed.2015.05.006.
101	Ishii, T. 2017. “The Ethics of Creating Genetically Modified Children Using Genome Editing.” *Curr. Opin. Endocrinol. Diabetes Obes.* 24 (6): 418–23. 10.1097/MED.0000000000000369.
102	Ishii, T., and I.D.M. Beriain. 2019. “Safety of Germline Genome Editing for Genetically Related ‘Future’ Children as Perceived by Parents.” *CRISPR J.* 2 (6): 370–75. 10.1089/crispr.2019.0010.
103	Ishii, T. 2017a. “Germ Line Genome Editing in Clinics: The Approaches, Objectives and Global Society.” *Brief Funct Genomics* 16 (1): 46–56. 10.1093/bfgp/elv053.
104	Ishii, T. 2017b. “Reproductive Medicine Involving Genome Editing: Clinical Uncertainties and Embryological Needs.” *Reprod Biomed Online* 34 (1): 27–31. 10.1016/j.rbmo.2016.09.009.
105	Jasanoff, S., J. B. Hurlbut, and K. Saha. 2019. “Democratic Governance of Human Germline Genome Editing.” *CRISPR J* 2 (5): 266–71. 10.1089/crispr.2019.0047.
106	Jedwab, A., D.F. Vears, C. Tse, and C. Gyngell. 2020. “Genetics Experience Impacts Attitudes towards Germline Gene Editing: A Survey of over 1500 Members of the Public.” *J. Hum. Genet.* 65 (12): 1055–65. 10.1038/s10038-020-0810-2.
107	Johnston, J. 2020. “Shaping the CRISPR Gene-Editing Debate: Questions About Enhancement and Germline Modification.” *Perspect Biol Med* 63 (1): 141–54. 10.1353/pbm.2020.0011.
108	Juengst, E.T. 2017. “Crowdsourcing the Moral Limits of Human Gene Editing?” *Hastings Cent Rep* 47 (3): 15–23. 10.1002/hast.701.
109	Juengst, E.T., G.E. Henderson, R.L. Walker, J.M. Conley, D. MacKay, K.M. Meagher, K. Saylor, M. Waltz, K.J. Kuczynski, and R.J. Cadigan. 2018. “Is Enhancement the Price of Prevention in Human Gene Editing?” *CRISPR J.* 1 (6): 351–54. 10.1089/crispr.2018.0040.
110	Kang, X.J., C. I. N. Caparas, B. S. Soh, and. Fan. 2017. “Addressing Challenges in the Clinical Applications Associated with CRISPR/Cas9 Technology and Ethical Questions to Prevent Its Misuse.” *Protein Cell* 8 (11): 791–95. 10.1007/s13238-017-0477-4.
111	Karagyaur, M.N., A.Y. Efimenko, P.I. Makarevich, P.A. Vasiluev, Z.A. Akopyan, E.V. Bryzgalina, and V.A. Tkachuk. 2019. “Ethical and Legal Aspects of Using Genome Editing Technologies in Medicine (Review).” *Sovrem. Tehnol. Med.* 11 (3): 117–32. 10.17691/stm2019.11.3.16.
112	Khan, S.H. 2019. “Genome-Editing Technologies: Concept, Pros, and Cons of Various Genome-Editing Techniques and Bioethical Concerns for Clinical Application.” *Mol. Ther. Nucl. Acids* 16: 326–34. 10.1016/j.omtn.2019.02.027.
113	Kleiderman, E., and U. Ogbogu. 2019. “Realigning Gene Editing with Clinical Research Ethics: What the ‘CRISPR Twins’ Debacle Means for Chinese and International Research Ethics Governance.” *Account Res* 26 (4): 257–64. 10.1080/08989621.2019.1617138.
114	Knoppers, B. M., and E. Kleiderman. 2019. “‘CRISPR Babies’: What Does This Mean for Science and Canada?” *CMAJ* 191 (4): E91–92. 10.1503/cmaj.181657.
115	Knoppers, B.M., and E. Kleiderman. 2019. “Heritable Genome Editing: Who Speaks for ‘Future’ Children?” *CRISPR J.* 2 (5): 285–92. 10.1089/crispr.2019.0019.
116	Kohn, D.B., M.H. Porteus, and A.M. Scharenberg. 2016. “Ethical and Regulatory Aspects of Genome Editing.” *Blood* 127 (21): 2553–60. 10.1182/blood-2016-01-678136.
117	Kratzer, K., L.J. Getz, T. Peterlini, J.-Y. Masson, and G. Dellaire. 2022. “Addressing the Dark Matter of Gene Therapy: Technical and Ethical Barriers to Clinical Application.” *Hum. Genet.* 141 (6): 1175–93. 10.1007/s00439-021-02272-5.
118	Krimsky, S. 2019a. “Breaking the Germline Barrier in a Moral Vacuum.” *Account Res* 26 (6): 351–68. 10.1080/08989621.2019.1644171.
119	Krimsky, S. 2019b. “The Moral Choices on CRISPR Babies.” *Am J Bioeth* 19 (10): 15–16. 10.1080/15265161.2019.1644824.
120	Krishan, K., T. Kanchan, and B. Singh. 2016. “Human Genome Editing and Ethical Considerations.” *Sci Eng Ethics* 22 (2): 597–99. 10.1007/s11948-015-9675-8.
121	Krishan, K., T. Kanchan, B. Singh, N. Baryah, and S. Puri. 2018. “Germline Editing: Editors Cautionary.” *Clin. Ter.* 169 (2): e58–59. 10.7417/T.2018.2053.
122	Labude, M.K., V. Xafis, P.S. Lai, and C. Mills. 2022. “Vulnerability and the Ethics of Human Germline Genome Editing.” *CRISPR J.* 5 (3): 358–63. 10.1089/crispr.2021.0053.
123	Lecuona, I.D., M. Casado, G. Marfany, M.L. Baroni, and M. Escarrabill. 2017. “Gene Editing in Humans: Towards a Global and Inclusive Debate for Responsible Research.” *Yale J. Biol. Med.* 90 (4): 673–81.
124	Ledford, H. 2019. “CRISPR Babies: When Will the World Be Ready?” *Nature* 570 (7761): 293–96. 10.1038/d41586-019-01906-z.
125	Lee, 2022 “The Crispr Revolution in Genome Engineering: Perspectives from Religious Ethics.” *J. Relig. Ethics*. 50: 333–360. 10.1111/jore.12402.
126	Lehmann, L.S. 2019. “Using the 4-S Framework to Guide Conversations With Patients About CRISPR.” *AMA J Ethics* 21 (12): E1029–35. 10.1001/amajethics.2019.1029.
127	Li, J.-R., S. Walker, J.-B. Nie, and X.-Q. Zhang. 2019. “Experiments That Led to the First Gene-Edited Babies: The Ethical Failings and the Urgent Need for Better Governance.” *J. Zhejiang Uni. Sci. B* 20 (1): 32–38. 10.1631/jzus.B1800624.
128	Liao, S.M. 2019. “Designing Humans: A Human Rights Approach.” *Bioethics* 33 (1): 98–104. 10.1111/bioe.12519.
129	Locke, L.G. 2020. “The Promise of CRISPR for Human Germline Editing and the Perils of ‘Playing God.’” *CRISPR J* 3 (1): 27–31. 10.1089/crispr.2019.0033.
130	Lorenzo, D., M. Esquerda, and Grupo Interdisciplinar en Bioética. 2019. “Map of Ethical Conflicts of the CRISPR-Cas9 Gene Edition Technique.” *Med Clin (Barc)* 153 (9): 357–59. 10.1016/j.medcli.2019.03.024.
131	Lovell-Badge, 2019. “CRISPR Babies: A View from the Centre of the Storm.” *Development* 146 (3): dev175778. 10.1242/dev.175778.
132	Lyon, J. 2017. “Bioethics Panels Open Door Slightly to Germline Gene Editing.” *JAMA* 318 (17): 1639–40. 10.1001/jama.2017.13962.
133	Macintosh, K.L. 2019. “Heritable Genome Editing and the Downsides of a Global Moratorium.” *CRISPR J* 2 (5): 272–79. 10.1089/crispr.2019.0016.
134	Malmqvist, E. 2021. “Clinical Trials of Germline Gene Editing: The Exploitation Problem.” *Bioethics* 35 (7): 688–95. 10.1111/bioe.12903.
135	Mandrioli, M. 2022. “Genome Editing among Bioethics and Regulatory Practices.” *Biomolecules* 12 (1):13. 10.3390/biom12010013.
136	Marchant, G.E. 2021. “Global Governance of Human Genome Editing: What Are the Rules?” *Annu Rev Genomics Hum Genet* 22: 385–405. 10.1146/annurev-genom-111320-091930.
137	Marinelli, S., and A. Del Rio. 2020. “Beginning of Life Ethics at the Dawn of a New Era of Genome Editing: Are Bioethical Precepts and Fast-Evolving Biotechnologies Irreconcilable?” *Clin. Ter.* 171 (5): e407–11. 10.7417/CT.2020.2249.
138	Mariscal, C., and A. Petropanagos. 2016. “CRISPR as a Driving Force: The Model T of Biotechnology.” *Monash Bioeth Rev* 34 (2): 101–16. 10.1007/s40592-016-0062-2.
139	Master, Z., and P. Bedford. 2018. “CRISPR Gene Editing Should Be Allowed in Canada, But Under What Circumstances?” *J. Obstet. Gynaecol. Can.* 40 (2): 224–26. 10.1016/j.jogc.2017.08.028.
140	Mathews, D.J.H. 2018. “Solidarity in the Age of CRISPR.” *CRISPR J.* 1 (4): 261–63. 10.1089/crispr.2018.29026.mat.
141	Matthews, K. R. W., and A.S. Iltis. 2019. “Are We Ready to Genetically Modify a Human Embryo? Or Is It Too Late to Ask?” *Account Res* 26 (4): 265–70. 10.1080/08989621.2019.1617139.
142	McCaughey, T., P. G. Sanfilippo, G.E. C. Gooden, D.M. Budden, L. Fan, E. Fenwick, G. Rees, et al. 2016. “A Global Social Media Survey of Attitudes to Human Genome Editing.” *Cell Stem Cell* 18 (5): 569–72. 10.1016/j.stem.2016.04.011.
143	McConnell, S.C. 2019. “An Exclusive Interview With CRISPR.” *AMA J Ethics* 21 (12): E1079–88. 10.1001/amajethics.2019.1079.
144	Meagher, K.M., and Z. Master. 2019. “Fostering a Prevention Mindset for Responsible Gene Editing.” *Account Res* 26 (4): 251–56. 10.1080/08989621.2019.1617140.
145	Melo-Martín, I. de, and Z. Rosenwaks. 2021. “Human Embryo Genetic Editing: Hope or Pipe Dream?” *Fertil. Steril.* 116 (1): 25–26. 10.1016/j.fertnstert.2021.04.017.
146	Memi, F., A. Ntokou, and I. Papangeli. 2018. “CRISPR/Cas9 Gene-Editing: Research Technologies, Clinical Applications and Ethical Considerations.” *Semin. Perinatol.* 42 (8): 487–500. 10.1053/j.semperi.2018.09.003.
147	Miguel Beriain, I. de, and B. Sanz. 2020. “Human Dignity and Gene Editing: Additional Support for Raposo’s Arguments.” *J Bioeth Inq* 17 (2): 165–68. 10.1007/s11673-020-09969-8.
148	Müller, M., M. Schneider, M. Salathé, and E. Vayena. 2020. “Assessing Public Opinion on Crispr-Cas9: Combining Crowdsourcing and Deep Learning.” *J. Med. Internet Res.* 22 (8): e17830. 10.2196/17830.
149	Mulvihill, J.J., B. Capps, Y. Joly, T. Lysaght, H.A.E. Zwart, and R. Chadwick. 2017. “Ethical Issues of CRISPR Technology and Gene Editing through the Lens of Solidarity.” *Br. Med. Bull.* 122 (1): 17–29. 10.1093/bmb/ldx002.
150	Napoletano, S., V. Piersanti, and G. Rallo. 2021. “CRISPR -Cas9: A Groundbreaking New Technique Which Ushers in New Prospects and Just as Many Doubts.” *Clin Ter* 171 (1): e52–54. 10.7417/CT.2021.2281.
151	Nestor, M.W., and R.L. Wilson. 2020. “Beyond Mendelian Genetics: Anticipatory Biomedical Ethics and Policy Implications for the Use of CRISPR Together with Gene Drive in Humans.” *J Bioeth Inq* 17 (1): 133–44. 10.1007/s11673-019-09957-7.
152	Nie, J.-B. 2020. “Human Genome Editing and a Global Socio-Bioethics Approach.” *Hastings Cent Rep* 50 (6): 44–45. 10.1002/hast.1200.
153	Niemiec, E., and H.C. Howard. 2020a. “Ethical Issues Related to Research on Genome Editing in Human Embryos.” *Comput. Struct. Biotechnol. J.* 18: 887–96. 10.1016/j.csbj.2020.03.014.
154	Niemiec, E., and H.C. Howard. 2020b. “Germline Genome Editing Research: What Are Gamete Donors (Not) Informed about in Consent Forms?” *CRISPR J.* 3 (1): 52–63. 10.1089/crispr.2019.0043.
155	Noll, S. 2021. “Altered Inheritance: CRISPR and the Ethics of Human Genome Editing.” *Int. J. Fem. Approaches Bioeth.* 14 (1): 168–71. 10.3138/ijfab-14.1.br04.
156	Opar, A. 2019. “CRISPR-Edited Babies Arrived, and Regulators Are Still Racing to Catch Up.” *Nat Med* 25 (11): 1634–36. 10.1038/s41591-019-0641-x.
157	Ormond, K.E., Y. Bombard, V.L. Bonham, L. Hoffman-Andrews, H. Howard, R. Isasi, K. Musunuru, K.A. Riggan, M. Michie, and M. Allyse. 2019. “The Clinical Application of Gene Editing: Ethical and Social Issues.” *Personalized Med.* 16 (4): 337–50. 10.2217/pme-2018-0155.
158	Ormond, K.E., D.P. Mortlock, D.T. Scholes, Y. Bombard, L.C. Brody, W.A. Faucett, N.A. Garrison, et al. 2017. “Human Germline Genome Editing.” *Am. J. Hum. Genet.* 101 (2): 167–76. 10.1016/j.ajhg.2017.06.012.
159	Padden, C., and J. Humphries. 2020. “Who Goes First? Deaf People and CRISPR Germline Editing.” *Perspect Biol Med* 63 (1): 54–65. 10.1353/pbm.2020.0004.
160	Palacios-González, C. 2021. “Reproductive Genome Editing Interventions Are Therapeutic, Sometimes.” *Bioethics* 35 (6): 557–62. 10.1111/bioe.12846.
161	Parent, B. & A. Turi. 2019. “Nature beyond Control: How Expectations Should Inform Decisions about Human Germline Engineering.” *J Assist Reprod Genet* 36 (9): 1771–77. 10.1007/s10815-019-01528-4.
162	Pavlovic, Z.J., M.R. Sax, A.S. Kim, and A.H. DeCherney. 2020. “Altered Evolution: Are Reproductive Endocrinology and Infertility Specialists Ready for the Genetically Engineered Future?” *J. Assisted Reprod. Genet.* 37 (12): 2949–54. 10.1007/s10815-020-01963-8.
163	Peng, Y., J. Lv, L. Ding, X. Gong, and Q. Zhou. 2022. “Responsible Governance of Human Germline Genome Editing in China(Dagger).” *Biol. Reprod.* 107 (1): 261–68. 10.1093/biolre/ioac114.
164	Piergentili, R., A. Del Rio, F. Signore, F. Umani Ronchi, E. Marinelli, and S. Zaami. 2021. “Crispr-Cas and Its Wide-Ranging Applications: From Human Genome Editing to Environmental Implications, Technical Limitations, Hazards and Bioethical Issues.” *Cells* 10 (5). 10.3390/cells10050969.
165	Ranisch, R. 2017. “Germline Genome Editing and the Functions of Consent.” *Am J Bioeth* 17 (12): 27–29. 10.1080/15265161.2017.1388875.
166	Ranisch, R. 2020. “Germline Genome Editing versus Preimplantation Genetic Diagnosis: Is There a Case in Favour of Germline Interventions?” *Bioethics* 34 (1): 60–69. 10.1111/bioe.12635.
167	Ranisch, R., T. Rudolph, H.-J. Cremer, and N. Knoepffler. 2020. “Ordo-Responsibility for Germline Gene Editing.” *CRISPR J.* 3 (1): 37–43. 10.1089/crispr.2019.0040.
168	Ranisch, R., K. Trettenbach, and G. Arnason. 2023. “Initial Heritable Genome Editing: Mapping a Responsible Pathway from Basic Research to the Clinic.” *Med Health Care Philos* 26 (4): 31–35, 10.1007/s11019-022-10115-x.
169	Raposo, V.L. 2019. “CRISPR-Cas9 and the Promise of a Better Future.” *Eur J Health Law* 26 (4): 308–29. 10.1163/15718093-12264438.
170	Reyes, A.P., and F. Lanner. 2017. “Towards a CRISPR View of Early Human Development: Applications, Limitations and Ethical Concerns of Genome Editing in Human Embryos.” *Development* 144 (1): 3–7. 10.1242/dev.139683.
171	Rosenbaum, L. 2019. “The Future of Gene Editing - Toward Scientific and Social Consensus.” *New Engl. J. Med.* 380 (10): 971–75. 10.1056/NEJMms1817082.
172	Rossant, J. 2018. “Gene Editing in Human Development: Ethical Concerns and Practical Applications.” *Development* 145(16): dev150888 10.1242/dev.150888.
173	Rufo, Fabrizio, and Antonella Ficorilli. 2019. “From Asilomar to Genome Editing: Research Ethics and Models of Decision.” *NanoEthics* 13 (3): 223–32. 10.1007/s11569-019-00356-1.
174	Rulli, T. 2019. “Reproductive CRISPR Does Not Cure Disease.” *Bioethics* 33 (9): 1072–82. 10.1111/bioe.12663.
175	Saha, K., J.B. Hurlbut, and S. Jasanoff. 2018. “Response to Beriain.” *Trends Biotechnol.* 36 (12): 1206–7. 10.1016/j.tibtech.2018.08.004.
176	Sand, M., A. L. Bredenoord, and K. R. Jongsma. 2019. “After the Fact-the Case of CRISPR Babies.” *Eur J Hum Genet* 27 (11): 1621–24. 10.1038/s41431-019-0459-5.
177	Savulescu, J., J. Pugh, T. Douglas, and C. Gyngell. 2015. “The Moral Imperative to Continue Gene Editing Research on Human Embryos.” *Protein Cell* 6 (7): 476–79. 10.1007/s13238-015-0184-y.
178	Scheufele, D.A., M.A. Xenos, E.L. Howell, K.M. Rose, D. Brossard, and B.W. Hardy. 2017. “U.S. Attitudes on Human Genome Editing.” *Science* 357 (6351): 553–54. 10.1126/science.aan3708.
179	Schleidgen, S., H.-G. Dederer, S. Sgodda, S. Cravcisin, L. Lüneburg, T. Cantz, and T. Heinemann. 2020. “Human Germline Editing in the Era of CRISPR-Cas: Risk and Uncertainty, Inter-Generational Responsibility, Therapeutic Legitimacy.” *BMC Med Ethics* 21 (1): 87. 10.1186/s12910-020-00487-1.
180	Schuijff, M., M.D.T. De Jong, and A.M. Dijkstra. 2021. “A Q Methodology Study on Divergent Perspectives on CRISPR-Cas9 in the Netherlands.” *BMC Med Ethics* 22 (1): 48. 10.1186/s12910-021-00615-5.
181	Schwartz, S.A. 2017. “The Oncoming Challenge of Homo Superior.” *Explore (NY)* 13 (6): 364–66. 10.1016/j.explore.2017.09.004.
182	Schweikart, S.J. 2019. “What Is Prudent Governance of Human Genome Editing?” *AMA J Ethics* 21 (12): E1042–48. 10.1001/amajethics.2019.1042.
183	Scott, C. T. & C. Selin. 2019. “What to Expect When Expecting CRISPR Baby Number Four.” *Am J Bioeth* 19 (3): 7–9. 10.1080/15265161.2018.1562793.
184	Scott, R. & S. Wilkinson. 2017. “Germline Genetic Modification and Identity: The Mitochondrial and Nuclear Genomes.” *Oxf. J. Legal Stud.* 37 (4): 886–915. 10.1093/ojls/gqx012.
185	Seibert, D. 2019. “Gene Editing: The Power and Risks of Creation.” *J Am Assoc Nurse Pract* 31 (2): 85–88. 10.1097/JXX.0000000000000196.
186	Shanahan, M.A., K.M. Aagaard, L.B. McCullough, F.A. Chervenak, and A.A. Shamshirsaz. 2021. “Society for Maternal-Fetal Medicine Special Statement: Beyond the Scalpel: In Utero Fetal Gene Therapy and Curative Medicine.” *Am. J. Obstet. Gynecol.* 225 (6): B9–18. 10.1016/j.ajog.2021.09.001.
187	Shaw, D. 2020. “The Consent Form in the Chinese CRISPR Study: In Search of Ethical Gene Editing.” *J Bioeth Inq* 17 (1): 5–10. 10.1007/s11673-019-09953-x.
188	Sherkow, J.S., E.Y. Adashi, and I.G. Cohen. 2021. “Governing Human Germline Editing through Patent Law.” *JAMA* 326 (12): 1149–50. 10.1001/jama.2021.13824.
189	Shozi, B. 2020. “A Critical Review of the Ethical and Legal Issues in Human Germline Gene Editing: Considering Human Rights and a Call for an African Perspective.” *S. Afr. J. Bioeth. Law* 13 (1): 62–67. 10.7196/SAJBL.2020.v13i1.709.
190	Shozi, B. 2021. “Does Human Germline Genome Editing Violate Human Dignity? An African Perspective.” *J. Law Biosci.* 8 (1): lsab002. 10.1093/jlb/lsab002.
191	Simonstein, F. 2019. “Gene Editing, Enhancing and Women’s Role.” *Sci Eng Ethics* 25 (4): 1007–16. 10.1007/s11948-017-9875-5.
192	Singh, S.M. 2019. “Lulu and Nana Open Pandora’s Box Far beyond Louise Brown.” *CMAJ* 191 (23): E642–43. 10.1503/cmaj.71979.
193	Šlesingerová, E. 2019. “In Risk We Trust/Editing Embryos and Mirroring Future Risks and Uncertainties.” *Med Health Care Philos* 22 (2): 191–200. 10.1007/s11019-018-9851-0.
194	Smith, K. 2020. “Time to Start Intervening in the Human Germline? A Utilitarian Perspective.” *Bioethics* 34 (1): 90–104. 10.1111/bioe.12691.
195	Snure Beckman, E., N. Deuitch, M. Michie, M.A. Allyse, K.A. Riggan, and K.E. Ormond. 2019. “Attitudes Toward Hypothetical Uses of Gene-Editing Technologies in Parents of People with Autosomal Aneuploidies.” *CRISPR J.* 2 (5): 324–30. 10.1089/crispr.2019.0021.
196	Sparrow, R. 2019. “Yesterday’s Child: How Gene Editing for Enhancement Will Produce Obsolescence-and Why It Matters.” *Am J Bioeth* 19 (7): 6–15. 10.1080/15265161.2019.1618943.
197	Sugarman, J. 2015. “Ethics and Germline Gene Editing.” *EMBO Rep.* 16 (8): 879–80. 10.15252/embr.201540879.
198	Šuleková, M., and K.T. Fitzgerald. 2019. “Can the Thought of Teilhard de Chardin Carry Us Past Current Contentious Discussions of Gene Editing Technologies?” *Camb Q Healthc Ethics* 28 (1): 62–75. 10.1017/S0963180118000397.
199	Sykora, P., and A. Caplan. 2017. “The Council of Europe Should Not Reaffirm the Ban on Germline Genome Editing in Humans.” *EMBO Rep.* 18 (11): 1871–72. 10.15252/embr.201745246.
200	Taguchi, I., T. Yamada, R. Akaishi, I. Imoto, K. Kurosawa, K. Nakatani, F. Nomura, et al. 2019. “Attitudes of Clinical Geneticists and Certified Genetic Counselors to Genome Editing and Its Clinical Applications: A Nation-Wide Questionnaire Survey in Japan.” *J. Hum. Genet.* 64 (9): 945–54. 10.1038/s10038-019-0635-z.
201	“Take Stock of Research Ethics in Human Genome Editing.” 2017. *Nature* 549 (7672): 307. 10.1038/549307a.
202	Thaldar, D., M. Botes, B. Shozi, B. Townsend & J. Kinderlerer. 2020. “Human Germline Editing: Legal-Ethical Guidelines for South Africa.” *S. Afr. J. Sci.* 116 (9–10): 27–33. 10.17159/sajs.2020/6760.
203	Thaldar, D. & B. Shozi. 2020. “Procreative Non-Maleficence: A South African Human Rights Perspective on Heritable Human Genome Editing.” *CRISPR J* 3 (1): 32–36. 10.1089/crispr.2019.0036.
204	The Lancet. 2017a. “Genome Editing: Science, Ethics, and Public Engagement.” *Lancet* 390 (10095): 625. 10.1016/S0140-6736(17)32209-2.
205	The Lancet. 2017b. “Safeguarding the Future of Human Gene Editing.” *Lancet* 389 (10070): 671. 10.1016/S0140-6736(17)30389-6.
206	The Lancet. 2018. “Genome Editing: Proceed with Caution.” *Lancet* 392 (10144): 253. 10.1016/S0140-6736(18)31653-2.
207	Thompson, 2019. “How Should ‘CRISPRed’ Babies Be Monitored Over Their Life Course to Promote Health Equity?” *AMA J Ethics* 21 (12): E1036–41. 10.1001/amajethics.2019.1036.
208	Tilburt, J.C. 2020. “CRISPR Transgressions, the Language of Wrongness, and the Task of Ethics.” *Mayo Clin. Proc.* 95 (2): 221–23. 10.1016/j.mayocp.2019.12.026.
209	Tomlinson, T. 2018. “A Crispr Future for Gene-Editing Regulation: A Proposal for an Updated Biotechnology Regulatory System in an Era of Human Genomic Editing.” *Fordham Law Rev* 87 (1): 437–83.
210	Torrance, A.W. 2017. “CRISPR Becomes Clearer.” *Hastings Cent Rep* 47 (5): 5–6. 10.1002/hast.762.
211	Townsend, B.A. 2020. “Human Genome Editing: How to Prevent Rogue Actors.” *BMC Med Ethics* 21 (1): 95. 10.1186/s12910-020-00527-w.
212	Travis, J. 2015. “Genetic Engineering: Germline Editing Dominates DNA Summit.” *Sci.* 350 (6266): 1299–1300. 10.1126/science.350.6266.1299.
213	Turocy, J., E.Y. Adashi, and D. Egli. 2021. “Heritable Human Genome Editing: Research Progress, Ethical Considerations, and Hurdles to Clinical Practice.” *Cell* 184 (6): 1561–74. 10.1016/j.cell.2021.02.036.
214	Udwadia, F., and S. Singh. 2019. “Starting the Conversation: CRISPR’s Role in India.” *Indian J Med Ethics* 4 (NS) (4): 300–303. 10.20529/IJME.2019.016.
215	Van Baalen, S., J. Gouman, D. Houtman, B. Vijlbrief, S. Riedijk, and P. Verhoef. 2021. “The DNA-Dialogue: A Broad Societal Dialogue about Human Germline Genome Editing in the Netherlands.” *CRISPR J.* 4 (4): 616–25. 10.1089/crispr.2021.0057.
216	Vasiliou, Stella K., Eleftherios P. Diamandis, George M. Church, Henry T. Greely, Françoise Baylis, Charis Thompson, and Gerold Schmitt-Ulms. 2016. “CRISPR-Cas9 System: Opportunities and Concerns.” *Clin Chem* 62 (10): 1304–11. 10.1373/clinchem.2016.263186.
217	Veit, W., J. Anomaly, N. Agar, P. Singer, D.S. Fleischman, and F. Minerva. 2021. “Can ‘eugenics’ Be Defended?” *Monash Bioeth Rev* 39 (1): 60–67. 10.1007/s40592-021-00129-1.
218	Walters, L.R., R.M. Cook-Deegan, and E.Y. Adashi. 2021. “Governing Heritable Human Genome Editing: A Textual History and a Proposal for the Future.” *CRISPR J.* 4 (4): 469–76. 10.1089/crispr.2021.0043.
219	Wareham, C.S. 2020. “Genome Editing for Longer Lives: The Problem of Loneliness.” *J Bioeth Inq* 17 (2): 309–14. 10.1007/s11673-020-09967-w.
220	Zhang, D., A. Hussain, H. Manghwar, K. Xie, S. Xie, S. Zhao, R.M. Larkin, P. Qing, S. Jin, and F. Ding. 2020. “Genome Editing with the CRISPR-Cas System: An Art, Ethics and Global Regulatory Perspective.” *Plant Biotechnol J* 18 (8): 1651–69. 10.1111/pbi.13383.
221	Zhang, D., and R.K. Lie. 2018. “Ethical Issues in Human Germline Gene Editing: A Perspective from China.” *Monash Bioeth Rev* 36 (1–4): 23–35. 10.1007/s40592-018-0091-0.
222	Zhou, Q., Y. Zhang, Y. Zou, T. Yin, and J. Yang. 2020. “Human Embryo Gene Editing: God’s Scalpel or Pandora’s Box?” *Brief. Funct. Genomics* 19 (3): 154–63. 10.1093/bfgp/elz025.
223	Zimbelman, J.A. n.d. “Managing New Technology When Effective Control Is Lost: Facing Hard Choices With Crispr.” *J. Relig. Ethics* 50 (3): 433-60. 10.1111/jore.12406.
